# Supplementary material for: High-speed scanless entire bandwidth mid-infrared chemical imaging
Source: Nat Commun. 2023 Jul 4;14:3929. doi: 10.1038/s41467-023-39628-6 (PMC10319884; doi:10.1038/s41467-023-39628-6)
Supplement: Supplementary file 1 — Supplementary Information [file 41467_2023_39628_MOESM1_ESM.pdf]

Supplementary Information for

# High-speed scanless entire bandwidth mid-infrared chemical imaging


Yue Zhao [1,5,*], Shota Kusama [1], Yuji Furutani [2,3], Wei-Hong Huang [4], Chih-Wei Luo [4], Takao Fuji [1,†]

[1] *Laser Science Laboratory, Toyota Technological Institute, 2–12–1 Hisakata, Tempaku-ku, Nagoya 468–8511, Japan.*
[2] *Department of Life Science and Applied Chemistry, Nagoya Institute of Technology, Showa-Ku, Nagoya 466-8555, Japan.*
[3] *Optobiotechnology Research Center, Nagoya Institute of Technology, Showa-Ku, Nagoya 466-8555, Japan.*
[4] *Department of Electrophysics, National Yang Ming Chiao Tung University, Hsinchu 30010, Taiwan.*
[5] *Current address: Graduate School of Engineering College of Design and Manufacturing Technology, Muroran Institute of Technology, 27-1 Mizumoto-cho, Muroran, Hokkaido 050-8585, Japan.*

* e-mail: zhaoyue@muroran-it.ac.jp
† e-mail: fuji@toyota-ti.ac.jp


**S1. Hyperspectral images by skipping pixel rows.**

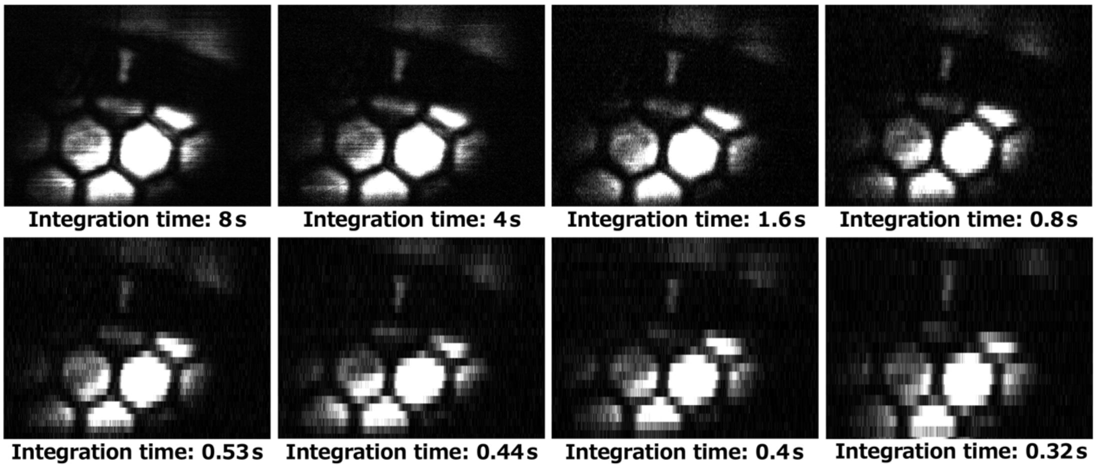


**Fig. S1.** Hyperspectral images by skipping pixel rows. The upper left image (640 × 480 pixels) was taken without any pixel row skips and the integration time was 8 s. Subsequent images were taken by skipping pixel rows, which are skipping 2 (integration time: 4 s), 5 (integration time: 1.6 s), 10 (integration time: 0.8 s), 15 (integration time: 0.53 s), 18 (integration time: 0.44 s), 20 (integration time: 0.4 s), and 25 (integration time: 0.32 s) pixel rows, respectively.

**S2. GaSe scotch tape film.**

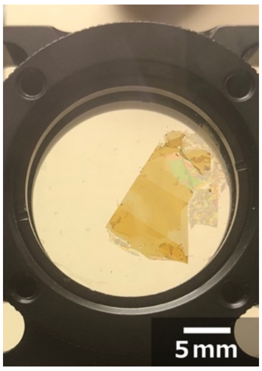


**Fig. S2.** GaSe scotch tape film.

**S3. The computer-aided design (CAD) of the microfluidic device.**

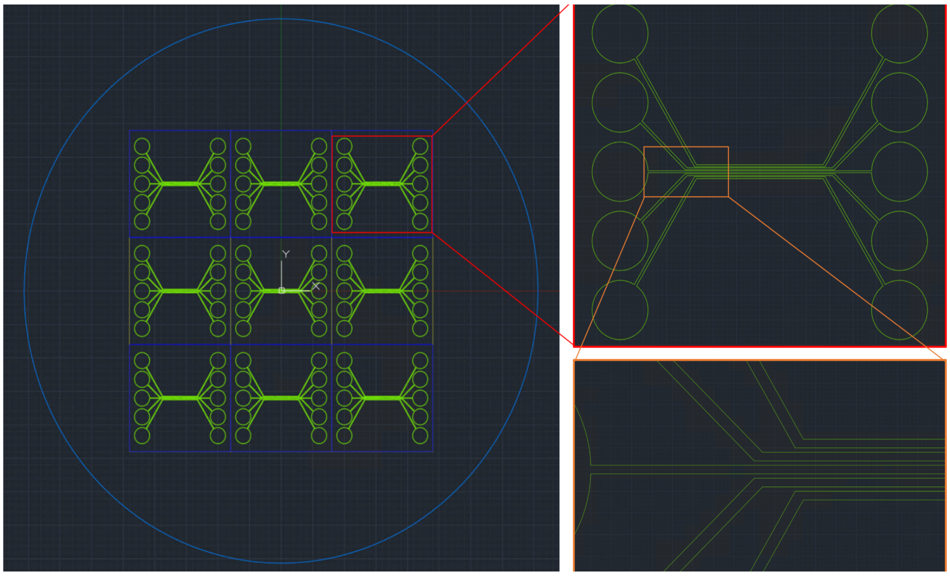

**Fig. S3.** The computer-aided design (CAD) of the microfluidic device. The depth of the microchannel was designed to be 25 μm.

**S4. The photograph of the microfluidic device.**

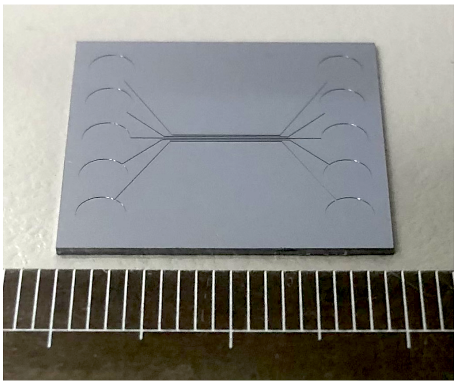

**Fig. S4.** The photograph of the microfluidic device. Each tick of the scale bar in the photo is 1 mm. The microchannel utilizes the capillary phenomenon that after the liquid is dropped into the circular groove, it will be sucked into the microchannel under the action of surface tension.
