# Supplementary material for: High-speed scanless entire bandwidth mid-infrared chemical imaging
Source: Nat Commun. 2023 Jul 4;14:3929. doi: 10.1038/s41467-023-39628-6 (PMC10319884; doi:10.1038/s41467-023-39628-6)
Supplement: Supplementary file 3 — Description of Additional Supplementary Files Template [file 41467_2023_39628_MOESM3_ESM.pdf]

Description of Additional Supplementary Files

File Name: Supplementary Movie 1
Description: A movie of the 2D image while the wavelength is continuously scanned over the entire mid-infrared bandwidth.

File Name: Supplementary Movie 2
Description: Movie of the water evaporation behavior captured with the high-speed imaging at a fixed wavenumber of 1640 cm^{-1} with a bandwidth of 50 cm^{-1}. Water was filled into three microchannels with a width of 200 µm and depth of 25 µm. In the middle channel, water starts to evaporate from the top and the water flows due to surface tension. The water in the left and right channels did not evaporate, thus the MIR was absorbed all the time and shows a dark distribution. The MIR transmit well at the position after the water flowed away, thus showing a brighter distribution. The time resolution is 0.2 ms and the exposure time is 1 µs per frame. The size of the movie is 1200 µm × 900 µm (640 × 480 pixels). The bit depth of the high-speed imaging is 8 bit.
