# Supplementary material for: High-speed scanless entire bandwidth mid-infrared chemical imaging
Source: Nat Commun. 2023 Jul 4;14:3929. doi: 10.1038/s41467-023-39628-6 (PMC10319884; doi:10.1038/s41467-023-39628-6)
Supplement: Supplementary file 6 — Reporting summary [file 41467_2023_39628_MOESM6_ESM.pdf]

nature portfolio

Corresponding author(s): Yue Zhao, Takao Fuji

Last updated by author(s): May 19, 2023

Reporting Summary

Nature Portfolio wishes to improve the reproducibility of the work that we publish. This form provides structure for consistency and transparency in reporting. For further information on Nature Portfolio policies, see our Editorial Policies and the Editorial Policy Checklist.

Statistics

For all statistical analyses, confirm that the following items are present in the figure legend, table legend, main text, or Methods section.

n/a	Confirmed	
☐	☒	The exact sample size (*n*) for each experimental group/condition, given as a discrete number and unit of measurement
☐	☒	A statement on whether measurements were taken from distinct samples or whether the same sample was measured repeatedly
☒	☐	The statistical test(s) used AND whether they are one- or two-sided<br>*Only common tests should be described solely by name; describe more complex techniques in the Methods section.*
☒	☐	A description of all covariates tested
☒	☐	A description of any assumptions or corrections, such as tests of normality and adjustment for multiple comparisons
☐	☒	A full description of the statistical parameters including central tendency (e.g. means) or other basic estimates (e.g. regression coefficient) AND variation (e.g. standard deviation) or associated estimates of uncertainty (e.g. confidence intervals)
☒	☐	For null hypothesis testing, the test statistic (e.g. *F*, *t*, *r*) with confidence intervals, effect sizes, degrees of freedom and *P* value noted<br>*Give P values as exact values whenever suitable.*
☒	☐	For Bayesian analysis, information on the choice of priors and Markov chain Monte Carlo settings
☒	☐	For hierarchical and complex designs, identification of the appropriate level for tests and full reporting of outcomes
☒	☐	Estimates of effect sizes (e.g. Cohen's *d*, Pearson's *r*), indicating how they were calculated

Our web collection on statistics for biologists contains articles on many of the points above.

Software and code

Policy information about availability of computer code

Data collection	1. LabVIEW, ver.19.0.1f1, National Instruments Corporation.<br>2. OPwave+, ver.4.17,Ocean Photonics.<br>3. SISHCapture_0.15nm(1069ch,610-770nm) , ver.1.1, EBA JAPAN CO.,LTD.<br>4. Phantom Camera Control (PCC) , ver.3.5, Vision Research.<br>5. Tabletop SEM TM4000 series, operation ver.1.10, control ver.1.7, stage unit ver.1.1, Hitachi High-Tech Corporation.<br>6. Spectra Manager, ver.2.10.01, JASCO Corporation.
Data analysis	1. MATLAB R2023a, ver.9.14.0, The MathWorks, Inc.<br>2. Spyder (Python), ver.3.10.9, Spyder developer community.<br>3. HSAnalyzer, ver.1.1, EBA JAPAN CO.,LTD.<br>4. Phantom Camera Control (PCC) , ver.3.5, Vision Research.<br>5. Spectra Manager, ver.2.10.01, JASCO Corporation.<br>6. ImageJ, ver.1.53 , Wayne Rasband.

For manuscripts utilizing custom algorithms or software that are central to the research but not yet described in published literature, software must be made available to editors and reviewers. We strongly encourage code deposition in a community repository (e.g. GitHub). See the Nature Portfolio guidelines for submitting code & software for further information.

Data

Policy information about availability of data

All manuscripts must include a data availability statement. This statement should provide the following information, where applicable:

- Accession codes, unique identifiers, or web links for publicly available datasets
- A description of any restrictions on data availability
- For clinical datasets or third party data, please ensure that the statement adheres to our policy

All data generated in this study have been deposited in the figshare database under accession code https://doi.org/10.6084/m9.figshare.22849559.

Research involving human participants, their data, or biological material

Policy information about studies with human participants or human data. See also policy information about sex, gender (identity/presentation), and sexual orientation and race, ethnicity and racism.

Reporting on sex and gender	N/A
Reporting on race, ethnicity, or other socially relevant groupings	N/A
Population characteristics	N/A
Recruitment	N/A
Ethics oversight	N/A

Note that full information on the approval of the study protocol must also be provided in the manuscript.

Field-specific reporting

Please select the one below that is the best fit for your research. If you are not sure, read the appropriate sections before making your selection.

☒ Life sciences ☐ Behavioural & social sciences ☐ Ecological, evolutionary & environmental sciences

For a reference copy of the document with all sections, see nature.com/documents/nr-reporting-summary-flat.pdf

Life sciences study design

All studies must disclose on these points even when the disclosure is negative.

Sample size	The experiment conducted in this study serves as a demonstration of the instrument we have developed. The samples involved in life sciences are A. cepa bulb leaves epidermal cells and mouse embryo sagittal plane. The A. cepa bulb leaf epidermal cells consist of more than 20 individual samples, while the mouse embryo sagittal plane comprises more than 5 individual samples. The sample size is determined based on our experimental observations and empirically derived numbers that ensure the production of reliable and reproducible results.
Data exclusions	No data is excluded.
Replication	To ensure robust reproducibility:<br>The experiment of Fig. 5 was repeated 8 times independently with similar results.<br>The experiment of Fig. 6 was repeated 7 times independently with similar results.
Randomization	All assignments were randomized. Onions were purchased in six batches from three different supermarkets, with onions being randomly selected from the shelves. The mouse embryo sagittal plane was obtained from GenoStaff Co., Ltd.
Blinding	No blinding was implemented for the experiments in this study, as all presented results were derived from qualitative analysis using mid-infrared spectroscopy. Therefore, the findings are objective and unaffected by human bias.

Reporting for specific materials, systems and methods

We require information from authors about some types of materials, experimental systems and methods used in many studies. Here, indicate whether each material, system or method listed is relevant to your study. If you are not sure if a list item applies to your research, read the appropriate section before selecting a response.

Materials & experimental systems

n/a	Involved in the study	
☒	☐	Antibodies
☒	☐	Eukaryotic cell lines
☒	☐	Palaeontology and archaeology
☐	☒	Animals and other organisms
☒	☐	Clinical data
☒	☐	Dual use research of concern
☐	☒	Plants

Methods

n/a	Involved in the study	
☒	☐	ChIP-seq
☒	☐	Flow cytometry
☒	☐	MRI-based neuroimaging

Animals and other research organisms

Policy information about studies involving animals; ARRIVE guidelines recommended for reporting animal research, and Sex and Gender in Research

Laboratory animals	13.5 days age mouse embryo was used in this study.
Wild animals	Wild animals were not involved in this study.
Reporting on sex	Gender was not taken into consideration in this study since all presented results were obtained through qualitative analysis using mid-infrared spectroscopy. Therefore, the experimental findings remained independent of gender influences.
Field-collected samples	This study did not involve any field-collected samples.
Ethics oversight	Ethical review was not necessary for this study since no experiments involving live animals were conducted. Additionally, there were no other experimental designs implemented that required ethical review.

Note that full information on the approval of the study protocol must also be provided in the manuscript.

Dual use research of concern

Policy information about dual use research of concern

Hazards

Could the accidental, deliberate or reckless misuse of agents or technologies generated in the work, or the application of information presented in the manuscript, pose a threat to:

No	Yes	
☒	☐	Public health
☒	☐	National security
☒	☐	Crops and/or livestock
☒	☐	Ecosystems
☒	☐	Any other significant area

Experiments of concern

Does the work involve any of these experiments of concern:

No	Yes	
☒	☐	Demonstrate how to render a vaccine ineffective
☒	☐	Confer resistance to therapeutically useful antibiotics or antiviral agents
☒	☐	Enhance the virulence of a pathogen or render a nonpathogen virulent
☒	☐	Increase transmissibility of a pathogen
☒	☐	Alter the host range of a pathogen
☒	☐	Enable evasion of diagnostic/detection modalities
☒	☐	Enable the weaponization of a biological agent or toxin
☒	☐	Any other potentially harmful combination of experiments and agents
